# Supplementary material for: Are carbon emissions trading and green financial instruments synergistic? -Comprehensive quantitative research based on content analysis
Source: PLoS One. 2024 Mar 7;19(3):e0298601. doi: 10.1371/journal.pone.0298601 (PMC10919601; doi:10.1371/journal.pone.0298601)
Supplement: S1 Data — (ZIP) [file pone.0298601.s001.zip › Data/地级市Z_F工作报告.zip!/╔╜╢1⁄2/┴┘╥╩╩╨/└┤╫╘╣1⁄2╓┌║┼í╛┬φ┐╦╩2╛▌═°í┐_2015.docx]

各位代表：

现在，我代表市人民政府向大会作政府工作报告，请予审议，并请市政协委员和其他列席会议的同志提出意见。

一、2014年工作回顾

过去一年，在市委的正确领导下，全市上下认真贯彻落实中央和省委、省政府决策部署，牢牢把握稳中求进的总基调，积极应对困难，科学务实作为，全力抓好稳增长、促改革、调结构、惠民生各项工作，经济社会保持平稳健康发展的良好势头。全市实现生产总值3569.8亿元，增长10.1%；一般公共预算收入251亿元，增长16.2%；固定资产投资2826亿元，增长16.2%，社会消费品零售总额、进出口总额分别突破2000亿元和100亿美元，增长12.8%和14.4%，主要经济指标增幅位居全省前列。

（一）转型升级步伐加快。大力实施现代产业推进、骨干企业培植、国际商贸名城建设“三项计划”，迎难而上调存量，紧盯高端优增量，经济结构日趋合理。三次产业比例调整为9.5∶46.2∶44.3，服务业增加值占比、高新技术产业产值占比分别提高1.5和1.4个百分点；万元GDP能耗完成省政府下达的年度目标，累计完成“十二五”总任务的80%以上。工业质效同增。规模以上工业企业主营业务收入突破万亿大关，增长16.1%，利润、利税分别增长10%和11.4%；200家重点骨干企业实现产值3892亿元，增长15.3%；山东临工获省长质量奖和中国企业五星品牌，金锣价格指数填补国内同行业空白。服务业繁荣发展。临沂商城交易额达到2687亿元，增长28.2%；全市电子商务交易额826亿元，增长50.1%；旅游总收入469.7亿元，增长13.5%；物流总额2.4万亿元，增长11.6%；首届资本交易大会、第五届商博会成功举办，我市被评为省优质商贸物流服务基地，鸿儒文化集团成为全国版权示范园区，临沂软件园获批全省电子商务示范基地，临沂云计算中心获得国家IDC资质认证。农业稳中向好。新建高标准农田75万亩，粮食总产435万吨；新造林34.7万亩，新增市级标准化畜禽养殖场68个、优质农产品品牌84个，新认证“三品一标”110个，成功创建山东省出口农产品质量安全示范市。重点项目支撑有力。引建市外招商项目476个，到位资金480亿元，增长21.5%；新开工过亿元项目908个，完成投资1642亿元。73个过20亿元项目签约，一批重点产业项目落地建设。县域经济日益壮大。15个县区（开发区）中有12个一般公共预算收入过10亿元，兰山区综合实力居全国市辖区第52位，临沂经济技术开发区跨入国家级产业园区百强行列。

（二）城镇建设亮点纷呈。加快中心城区、县城、小城镇和农村新型社区“四级联动”，城镇化率达到54.3%。新一轮中心城区控规覆盖率达72%，建成区面积和人口分别达到210平方公里、200万人；县城和省级示范镇总体规划修编基本完成，建成区面积超过25平方公里的县城达到4个，19个小城镇被列为全国重点镇；建设农村新型社区70个。中心城区基础设施“十大工程”加快推进，139项路桥工程开工，东外环、沂河路—西外环立交桥和临工大桥建成通车；新增供热面积700万平方米、供气能力5亿立方米，新建改造供水、排水、污水管网和输电线路110公里、157公里、70公里、60公里，人均公园绿地面积13.7平方米；污水集中处理率达到94%，8个国家考核断面水质达标率为98.7%。在全国率先完成智慧城市时空信息云平台设计，城乡环卫一体化经验在全省推广，有5个县（区）被授予全省生态文明乡村建设工作先进县。新建改建公路2086公里，沂沭铁路建设和胶新铁路电气化改造等进展顺利，鲁南高铁客运专线成功获批，老区人民期待已久的“高铁梦”有望变为现实。

（三）综合改革纵深推进。财税体制改革方面，建立全口径预算基本框架，市级预算公开部门扩大到232个，专项转移支付项目压减57%。土地利用方面，累计盘活闲置低效用地5万多亩，12个县（区）全部通过省级节约集约用地考核。农村改革方面，土地承包经营权确权登记基本完成，集体土地所有权确权发证全面完成，新增农民专业合作社2076个、家庭农场1298户。金融改革方面，新引进银行2家，新设立村镇银行3家，新增齐鲁股权交易中心挂牌企业26家，金运通网络支付公司获得第三方支付牌照，社会融资总量和贷款余额分别突破1000亿元和3000亿元。科技创新方面，临沂应用科学城投入使用，新增省级研发平台24家、院士工作站15家，新认定高新技术企业53家、国家级守合同重信用企业8家，常林液压元件产业集聚区成为国家级产业集聚区。政策争取方面，综合保税区获准设立，临沂机场作为航空口岸获批临时对外开放，国家级出口木制品质量安全示范区通过验收，成为国家首批生态文明先行示范区和全省唯一的基层卫生综合改革联系点。

（四）民生质量持续改善。全力办好十件惠民实事，民生支出298亿元，占公共财政总支出的65.5%。居民收入较快增长，城镇居民人均可支配收入30345元，农民人均纯收入11629元，分别增长10.3%和11.9%。就业创业不断扩大，城镇新增就业13.3万人、农村劳动力转移就业18.6万人。社会保障提标扩面，城乡居民医保整合工作完成，城镇职工基本医疗保险统筹基金、大额医疗救助年度最高支付限额分别提高到18万元、50万元，低保、五保对象政策范围内年度医疗救助标准提高到1万元；对418个村实施整村脱贫，贫困人口减少20万人；养老服务设施达到877处、床位5万余张；开工保障性安居工程4.3万套，新建农房9万户、改造农村危房1万户；89.3万农村居民饮水安全问题得到解决；重度残疾人提前五年领取居民基本养老保险金，蝉联“中国公益慈善七星级城市”。公共服务水平进一步提高，临沂一中北校区、青少年综合实践基地国家示范项目建成使用，8个县（区）通过国家、省义务教育均衡发展督导评估验收，2所职业院校通过国家中职示范建设项目验收 诛仙小说；省级区域医疗中心正式运行，县级公立医院综合改革扎实推进，基本药物采购总量保持全省第一；临沂大剧院和全国首家东夷文化主题博物馆建成使用，现代柳琴戏《又是一年桃花开》入选山东“文艺精品工程”；新建农村社区综合文化服务中心300个、健身场点1000个，成功举办第三届沂河国际运动娱乐节，首获亚运会金牌2枚。群众性精神文明创建、民主法治建设深入推进，人口计生、食品药品监管、安全生产、社会治安和信访维稳等都取得较好成绩。

（五）政府作风明显改进。扎实开展党的群众路线教育实践活动，严格履行党风廉政建设责任制，认真落实中央八项规定，坚决整“四风”、转作风，政府系统为民务实清廉形象进一步树立。厉行勤俭节约，市级公务接待费下降28.8%，政府会议、发文和评比减少20%以上。强化简政放权，取消、下放市级行政审批事项121项，提前实现本届政府削减1/2的目标；113项工商登记前置审批改为后置，新登记市场主体8.6万户，实现“个转企”9956户，企业数量增长46.6%。坚持依法行政，自觉接受人大法律监督、政协民主监督和群众社会监督，人大代表建议和政协提案办结率100%。构建四级便民服务体系，“12345”市民服务热线受理来电142万余件，诉求办结率、满意率分别达98.9%和95.9%。第三次经济普查圆满完成，并被评为全国先进集体；“建市场商会、助推大市场繁荣”的做法成为全国工商联系统十大创新；国防动员、人民防空、民兵预备役、双拥共建工作全面加强，妇女儿童、老龄、红十字、民族、宗教、对台、外事、侨务、供销、防震减灾、气象、史志、档案、保密、盐务等工作都有了新进展。

各位代表，2014年是很不平凡的一年。在宏观经济下行压力加大、各种困难挑战增多的情况下，取得这些成绩实属不易。这是省委、省政府和市委正确领导的结果，是市人大、市政协监督支持的结果，是全市上下团结奋斗、苦干实干的结果。在此，我代表市政府，向广大干部群众，向人大代表、政协委员、各民主党派、工商联、人民团体和离退休老同志，向驻临人民解放军、武警官兵和中央、省属单位，向参与和支持临沂发展的各界朋友，表示衷心的感谢和崇高的敬意！

在肯定成绩的同时，我们也应清醒看到发展中存在的困难和问题：经济总量虽然较大，但人均占有较低；产业结构不够合理，传统产业比重较高，新兴产业和新型业态发展相对滞后；自主创新能力不强，投资结构偏重，发展新动力有待培育；结构调整阵痛显现，有些企业生产经营困难；县域经济发展不够均衡，园区经济规模不大，财政面临持续增收压力；城镇功能配套还不完善，管理水平亟需提高，大气、水、土壤等环境问题仍很突出，公共服务存在供给不足等现象；政府作风还有待改进，社会信用体系尚不健全，营商环境需要进一步优化，等等。对此，我们一定高度重视，认真研究并加以解决。二、2015年工作安排

今年是全面推进依法治国的开局之年，是全面深化改革的关键之年，也是全面完成“十二五”规划任务的收官之年，做好今年工作意义重大。今年政府工作总体要求是：全面贯彻党的十八大和十八届三中、四中全会精神，以习近平总书记系列重要讲话和视察山东及临沂重要讲话、重要批示精神为指引，以稳中求进为总基调，以提高经济发展质量和效益为中心，适应新常态、抢抓新机遇、展现新作为，加快建设实力临沂、活力临沂、生态临沂、红色临沂、法治临沂和幸福临沂，为在全面建成小康社会进程中“走在前列”奠定坚实基础。

主要预期目标是：经济平稳健康发展。生产总值增长10%左右；一般公共预算收入增长12%左右；固定资产投资增长16%左右；社会消费品零售总额增长12.5%左右；进出口总额增长12%左右。结构不断调整优化。服务业增加值占比、高新技术产业产值占比分别提高1个和1.5个百分点；全社会研发投入占生产总值比重达到2%以上。民生保障更加有力。城镇和农村居民人均可支配收入分别增长10%和11%左右；居民消费价格涨幅控制在3%左右；城镇登记失业率控制在4%以内。生态环境持续改善。全面完成省政府下达的年度节能减排约束性目标；保持水污染防治成效走在全省前列；痛下决心向大气污染宣战，务求取得决定性胜利。

做好今年工作，应牢牢把握好几个重大原则：

第一，必须按“走在前列”的要求谋划和推动工作。临沂人口面积均占全省九分之一，地位举足轻重，支撑山东的责任重大。推进富民强市，振兴沂蒙老区，我们使命在肩、责无旁贷。必须按“走在前列”的要求谋划思路、制定措施，在各领域各方面力争上游、当排头兵。必须以功成不必在我的境界，一任接着一任干，一张蓝图绘到底。要在战略目标上不动摇，确保如期实现市委提出的“与全省同步、比全国提前全面建成小康社会”的目标，圆满完成“十二五”规划各项任务。启动“十三五”规划编制工作。要在战略定位上不动摇，把临沂放在全国、全省发展大局中来比较，登高望远、高点对标，打造鲁南苏北区域性中心城市，力争综合实力早日进入全省第一方阵。要在战略重点上不动摇，全力以赴壮产业、强县域、兴商贸，坚定不移推进经济转型、城市转型，靠全面深化改革破解瓶颈制约，以创新驱动培育新的增长点，用扩大对外开放拓展发展新空间，使经济更富活力、社会更加公平、发展更可持续。

第二，必须强化质量效益导向，促进又好又快发展。发展是硬道理，是解决一切问题的关键。发展不足、人均偏低是临沂最大的市情。新常态下，解决发展中的矛盾和问题，必须保持一定的增长速度，继续做大经济总量，持续提高人均占有水平。只有始终保持一定速度，几年跃上一个新台阶，美好愿景才能变成生动现实，才能真正实现后发崛起。我们要的速度是实实在在、没有水分的速度，是有质量、有效益的速度。必须坚持稳中求进、进中求好、好中求快、能快则快，做到调速不减势、量增质更优，推动经济总量、发展质量、人均占有量“三量同增”，速度效益同步提高。

第三，必须依靠大开放大项目大产业带动科学跨越发展。对外开放牵引全局。回顾临沂的发展历程，每一次跨越发展，每一次成功转型，都离不开扩大开放这个根本动力。没有大开放就没有大发展，开放的程度和水平决定着发展的速度和效益 党组织。必须实施更加积极主动的开放战略，主动融入“一带一路”格局，在更大范围、更广领域、更高水平上推进对外开放。必须以大开放的姿态、全球化的视野，运筹引进大项目，培植提升大产业，以开放带动经济转型升级。必须统筹抓好各类项目，推动项目落地建设，加快实施鲁南高铁客运专线、临沂机场三期改扩建、岚罗高速、“两直”特高压电网建设、岸堤水库扩容及除险加固等重大基础设施项目，积极推进一批正在实施和将要落地的重要产业项目，积蓄临沂经济发展后劲。

第四，必须紧扣“四化同步”目标，推动区域经济统筹协调发展。“四化”同步发展是全面建成小康社会的必由之路。当前，临沂正处在工业化加速、信息化带动、城镇化提质、农业现代化深化的关键时期。必须赋予区域经济发展“四化同步”新内涵，坚持全市一体化布局、差异化定位、特色化发展。引导各级按照主体功能区规划，发挥优势、协调联动，让城市经济当好龙头、县域经济争先进位、园区经济对标赶超、镇域经济竞相发展、农村经济生态高效。必须统筹城乡发展，协调推进交通、环卫、流通等公共服务一体化，不断提高城镇化质量。必须坚持产城融合、多规合一，统筹基础建设、产业发展和人口布局，培植一批承载新产业、新业态、新技术的增长板块。规划建设好国家林产工业科技示范园区、临空经济区、高铁片区等，形成多点支撑、协调发展的良好格局。

第五，必须把保障改善民生作为各项工作的根本出发点和落脚点。只有始终关注民生、不断改善民生，才能凝聚起科学跨越发展的正能量，才能实现经济发展和民生改善的良性循环。必须时刻把群众安危冷暖放在心上，坚持问政、问需、问计于民，把百姓的难事当成政府的要事，把百姓的关注点作为政府工作的着力点，多谋民生之利，多解民生之忧，多办一些雪中送炭的实事，使改革发展成果更多更公平地惠及老区人民。

今年的工作，突出抓好六个方面：

（一）以开放促转调，建设实力临沂。坚持对内对外开放相促进、引资引智相融合、请进来和走出去相结合，全力构建开放型经济新体系，带动产业转型、结构优化，打造临沂经济升级版。要在招大引强中培育主导产业。舍得拿出好资源、好企业、好项目招商，瞄准国内外五百强和行业龙头企业，引进一批产业龙头项目，持续扩大投资，做大做强产业规模。对规划的先进装备制造、高端木业、食品加工等十大千亿级产业，要“敲开核桃”，逐个进行剖析，逐一制定转调方案，使其与世界产业发展趋势对接、与国内外市场需求对接、与国际同行业先进水平对接，引导产品向下游发展、链条向高端延伸、产能向先进转变，打造一批千亿级产业航母，力争十大产业总产值过万亿元。推进矿产资源整合提升，壮大发展矿业经济。要在战略合作中壮大骨干企业。引导骨干企业学习借鉴山东临工、金正大和常林集团等企业的经验，面向国内外高端科研院所和知名企业，多方寻求合作，力争两三年内一半以上的骨干企业都能引进一个具有较大影响力的战略合作伙伴，打造一个高层次职业化的经营管理团队，建设一个把握行业发展方向的技术研发体系，培植一个拥有市场话语权的著名品牌。实施“千企千项千亿”技改工程，着力扶持10家现有百亿级企业发展壮大，鼓励10家有潜力的企业向百亿级迈进，引建10个左右有望成长为百亿级企业的项目，力争三年内新增百亿级企业15家以上。坚持以质、以技取胜，争创驰名商标、著名商标和山东省名牌产品，争取专利申请和授权量年均增长15%以上。要在对接国际市场中推动商城繁荣。大力推进商城国际化，扎实开展省级、积极争取国家级国际贸易综合改革试点，推动临沂商城向“买全国、卖全球，买全球、卖全国”转变。围绕商贸与物流融合，加快编制市场空间布局规划和物流业中长期发展规划，出台加快现代流通业发展意见，搞好鲁南铁路物流园区、现代物流园区、航空物流园区和临港物流基地建设，搭建临沂市物流公共信息平台，引进国内外航空货运、快递物流等龙头企业，促进商贸、物流企业之间的战略合作和业务协同，打造物流枢纽城市和区域分拨中心。今年，临沂商城交易额要突破3000亿元，物流总额要达到2.6万亿元。围绕线上与线下融合，对传统批发市场进行电商化提升，推进20万平方米线上线下结合的新型市场项目建设，力争在30%的市场设立电商经营服务区，电子商务交易额突破1000亿元；实施骨干电商三年培育工程，发展电子商务园区10个、龙头企业50家、特色网商5000家以上，形成一批在全省有影响的电商镇、村；办好第六届商博会和第68届全国教育装备展等知名展会，壮大会展经济。围绕内贸与外贸融合，培育引进大型外贸经营主体，新增有权外贸企业500家；加快综合保税区建设，尽快通过封关验收；丰富临沂港港口功能，实行检验检疫监管快速放行模式；加强对外经贸联络与合作，在重要口岸城市和外资来源地派驻联络员、设立办事处，搭建国际贸易平台；完善跨国采购体系，办好中欧商贸物流合作园区，支持批发市场、龙头企业走出去，打造进口商品分销中心；学习借鉴上海自贸区经验，争取设立临沂商城国际贸易综合改革试验区，打造内外贸融合发展的试验田。同时，制定开发区转型升级方案，创新管理体制，提高投入产出、质量安全、科技含量等标准，实施一批高端产业项目。要在招才引智中强化创新驱动。面向全球广泛吸引高端人才，力争引进“千人计划”、“万人计划”专家等创新创业团队20个以上，带资金、技术、项目的人才100名以上。构建科技创新创业孵化体系，以临沂高新区、临沂经济技术开发区和临沂应用科学城为载体，规划建设开放共享的“众创空间”，力争科技企业孵化器面积达到100万平方米，新增科技型企业300家、高新技术企业50家。鼓励企业创建国家和省级重点实验室、工程技术研究中心、企业技术中心，力争新增国家级创新平台3家、省级20家。深化产学研协同创新，支持临沂大学等高校和职业院校建设创业大学和市级公共实训中心，服务现代产业发展。积极对接国家和省各类创新基金，用好用活科技投入政策，激发企业创新活力。

（二）以改革破难题，建设活力临沂。突出问题导向，尽快把属于地方事权、现实迫切需要、条件已具备的改革事项推开，促进大众创业、万众创新。深化行政管理体制改革。以“四张清单、一个平台”为主要架构，建立完善政府权力运行体系。进一步简政放权，再取消和下放一批行政审批事项，大幅度减少投资项目前置审批，全面清理非行政许可审批；推行“三证合一”改革，加强“先照后证”事中事后监管；完善四级便民服务网络，加快市民中心建设，开办市级政务服务网上大厅，建立统一规范的行政服务平台；推进行业协会、商会与政府部门脱钩，对中介服务进行清理、规范，全面优化营商环境。深化财税体制改革。政府收支一律纳入预算并公开，所有公共资金一律接受审计监督，对预算资金、重大投资、重点工程执行进度和效果实行全过程监督；长期闲置的沉淀资金一律调整回收，切实用到保民生、补短板、增后劲的“刀刃”上；对国有资产和企业一律从严监管，严格产权交易操作流程，坚决防止国有资产流失。深化投融资体制改革。探索政府购买服务、与社会资本合作等财政支持方式，逐步取消对竞争性行业的直接补助，管好用好市级产业引导基金，提高财政资金使用效率。实施县域金融“五个一”工程，择优引进各类金融机构，办好临沂国际商品交易中心，举办第二届资本交易大会，鼓励企业发行股票、债券，力争社会融资规模增长20%以上。深化企业改革。推动国有资产整合重组，完善以管资本为主的国有资产监管模式。充分发挥民营经济的主力军作用，鼓励发展混合所有制经济，支持非公有制企业进入特许经营领域、参与国有企业改制重组；深入实施“个转企、小升规、规改股、股上市”，推动1万户“个转企”，500家小微企业升级为规模以上企业，500家规模企业改制为股份有限公司，3家企业在境内外上市，15家企业在场外市场挂牌交易。深化农村改革。搞好确权登记扫尾验收和配套改革，探索土地承包经营权流转、抵押、担保等办法，建立市县农业企业融资担保中心，推动适度规模经营。引导发展耕地面积100亩以上的家庭农场，培育100家示范性家庭农场、100家农业产业化龙头企业、150家市级示范合作社和2万名新型职业农民。加快供销社改革，启动农田小水利产权改革。稳妥推进事业单位、县级公立医院、机关事业单位养老保险制度以及公务用车制度等改革，不断完善科学发展的体制机制。

（三）以大气污染治理为突破口，建设生态临沂。良好生态环境是重要的公共产品。要顺应人民群众从“盼温饱”到“盼环保”的新期待，用省级“两型”社会建设先行先试政策，推动我市国家生态文明先行示范区建设，探索绿色循环低碳发展之路，共建天蓝、地绿、水清的美好家园，实现生态与经济建设的互促共赢。铁腕治理雾霾。把开展大气污染防治攻坚行动作为民心工程，在治标上出重拳、治本上下功夫、督查上动真格、惩治上敢碰硬，确保全市空气质量改善幅度进入全省前六位、主要指标位次退出全省后三位、空气质量指数瞬时值退出全国排名末位。集中力量啃下工业污染这块“硬骨头”，依法限期治理412家、停产治理57家企业，停业关闭整治无望企业，全面清除土小企业；加快优化产业布局，对严重影响区域环境的企业，采取“退城进园”措施，有序实施关闭搬迁；抓好扬尘治理，对达不到标准要求的建筑工地立即停工整改，严禁渣土运输车辆抛洒遗漏；全面落实机动车尾气污染、餐饮油烟、秸秆禁烧等治理措施。对破坏大气环境质量的行为，露头就打、依法严惩，对责任落实不得力的地方和部门加大行政问责力度，齐心协力打赢治霾攻坚战。集约利用资源。严守耕地保护红线，争创全国国土资源节约集约模范市；搞好国家级新能源汽车示范城市建设，大力支持新能源汽车推广应用；着力发展循环经济和环保产业，力争2个国家级园区和30%以上省级园区完成循环化改造任务；积极应用绿色清洁能源，推动哈纳斯绿色能源推广中心项目落地，确保天然气使用量突破7亿立方米；创建国家可再生能源建筑应用示范市，完成示范面积200万平方米；落实水资源“三条红线”，加快实施祊河梯级开发等雨洪资源利用工程，万元工业增加值用水量要比2010年下降22%。完善生态系统。建立健全生态补偿机制，支持蒙阴县、罗庄区率先探索绿色生态发展和循环转型发展模式。推进沿路、沿河、沿水库、城郊和山岭造林绿化，提高森林覆盖率，创建一批国家园林县城。健全“治、用、保”流域治理体系，确保国家考核的8个断面和市控重点河流水质稳定达标，代表山东省迎接淮河流域水污染防治核查工作务求取得优异成绩，争取通过全国水生态文明城市建设试点验收。发展两型农业。围绕建设农产品质量安全放心市，打造资源节约、环境友好农业，进一步打响“生态沂蒙山、优质农产品”品牌。实施耕地质量提升计划，开展土壤污染治理与修复试点，有效减少农村面源污染。开展粮食高产创建，支持发展节水农业和水肥一体化，新增“旱能浇、涝能排”高标准农田60万亩，确保粮食安全。

（四）以弘扬沂蒙精神为主旋律，建设红色临沂。沂蒙精神是临沂的宝贵财富，是激励全市上下干事创业的强大动力。要把红色资源利用好、红色传统发扬好、红色基因传承好，扩大沂蒙文化的感召力和影响力。传承红色文化。加强基地建设，维护好沂蒙精神教育、宣传和纪念设施，改善党的群众路线教育基地办学条件，充分发挥沂蒙革命纪念馆等场所作用，吸引更多的群众接受红色文化的教育和熏陶。开展主题教育，在全市各行业各层面，培育树立弘扬沂蒙精神的先进典型，激励广大干部群众大力传承红色文化。增进军政军民团结，确保全国双拥模范城创建实现“四连冠”。深入挖掘文化资源，努力推出一批体现临沂区域特色、红色文化特点的精品力作。发展红色文化旅游，对沂蒙红色影视基地、八路军115师司令部暨省政府旧址、华东革命烈士陵园等红色景区进行提升完善，打造全国红色旅游修学基地；提升孟良崮战役纪念地景区，加快创建国家5A级红色旅游景区；发展以“沂蒙人家”为品牌的乡村旅游，打造市级生态旅游特色村100个、省级“好客人家”农家乐50家；搞好岱崮地貌等自然资源的保护开发，加快建设环蒙山休闲养生旅游度假目的地，打响“好客山东、亲情沂蒙”品牌。发掘传统文化。以“十大文化行动”为抓手，搞好书圣、智圣、算圣、孝圣等历史名人和汉晋文化古迹的保护和推介，抓好兵学博物馆建设，弘扬书法文化、兵学文化、孝悌文化等优秀传统文化。开展沂蒙传统村落普查认定，实施乡村记忆工程，逐步在每个镇建立历史纪念室，努力保护文化遗存和乡村风情风貌。大力发展文化产业，建设一批集聚区、新上一批大项目、培植一批骨干文化企业。办好书圣文化节和第二届中国（临沂）国际艺术大展，进一步塑造沂蒙文化品牌。繁荣基层文化。开展基层公共文化服务能力建设年活动，推进市县文化馆（艺术馆）、图书馆规范化建设，力争全市乡镇、街道综合文化站达到国家三级以上标准。按照“五个一”标准，建设300个社区综合文化服务中心。免费开放“三馆一站”，鼓励创办庄户剧团、社区艺术团，扎实开展送文化下乡，丰富群众精神文化生活。（五）以依法行政为先导，建设法治临沂。各级政府要全面落实依法治市要求，牢固树立法治思维，自觉把宪法和法律作为政府工作的根本准则，加快构建办事依法、遇事找法、解决问题用法、化解矛盾靠法的法治良序。依法科学民主决策。健全公众参与、专家论证、风险评估、合法性审查、集体讨论决定的重大决策机制，发挥政府法律顾问作用，完善协商民主制度，凡属“三重一大”事项都要充分发扬民主，确保政府决策合法；严格执行规范性文件制定计划，及时搞好“立改废释”工作，确保规范性文件合法有效。推进公正文明执法。健全行政执法人员培训制度，提高行政执法人员法律素质；建设行政处罚电子监察系统，对行政处罚运行实行全程、实时监控，提高执法效率，维护执法公正；在行政执法、行政事务和公共服务中，坚持程序公正和结果公正有机统一，坚决反对选择性执法、随意性执法等行为。探索建立执法全过程记录制度，依法公开权力运行流程等信息，让群众在一件件具体行政行为中切身感受到公平、公正。自觉接受各方监督。认真执行人大及其常委会决议、决定，坚持重大事项向人大报告、向政协通报制度，认真办理人大代表议案、建议和政协委员提案，广泛听取民主党派、工商联、无党派人士和人民团体意见，积极接受新闻舆论和社会监督；落实行政机关依法出庭应诉等制度，严肃查处政府系统渎职失职行为；落实行政执法责任和过错追究制，充分发挥行政复议监督纠错功能，推动政府系统依法、秉公、廉洁执法。建设法治社会。全面推进“六五”普法，加强普法志愿者队伍建设，重点搞好青少年和进城务工人员法治宣传教育，增强全民法治观念；扎实开展法律进机关、进乡村、进社区、进学校、进企业主题宣传教育活动，鼓励创建法治县（区）、法治村居和法制教育示范学校、单位，支持各类社会主体自我约束、自我管理；发挥市民公约、乡规民约、行业规章等社会规范在社会治理中的积极作用，支持和保障社会组织依法自律；搞好司法救助，依法运用调解、信访、行政复议、行政诉讼、民商事仲裁等多种手段化解纠纷，引导和支持群众通过法定程序理性表达诉求，进一步形成尊法学法守法用法的法治环境。

（六）以改善民生为落脚点，建设幸福临沂。把民之所望作为政之所向，带着感情和责任，全力抓好惠民实事，进一步增强广大人民群众的幸福感和满意度。促进创业就业。实施农民工职业技能提升、权益保障、公共服务三年行动计划，统筹推进高校毕业生、城镇困难人员、退役军人等重点群体就业；继续实施全民创业行动，力争每个县区建立1处创业孵化基地或创业园区，支持大学生创业就业；办好城区零工市场，形成“30分钟公共就业服务圈”；新增城镇就业8万人、农村劳动力转移就业15.5万人，引导不少于4万名外出务工人员返乡就业。织密社保网络。强化社保扩面征缴，力争城乡居民养老保险适龄参保率达到99%以上，基本医疗保险和城镇职工社保覆盖率，分别达到95%和90%以上。加大社会救助力度，所有县（区）开通医疗救助“一站式”即时结算，对重特大疾病患者实行有效救助；关注群众生命健康，加大应急救护知识普及力度；健全特殊困难群众生活救助与物价补贴动态机制，推进“助残奔康”和计生困难家庭扶助工程；建设各类保障性安居工程4.9万套(户)，实现城镇中低收入家庭住房应保尽保；搞好精准扶贫，力争再有20万人脱贫。提升养老服务水平，探索医疗机构参与发展全托养老院，加快建设市老年养护院、社会福利院，新建一批城市社区老年人照料中心和农村幸福院。优化公共服务。完善优质教育体系，新建标准幼儿园155所，改造农村义务教育校舍120万平方米；所有县（区）县域义务教育均衡发展，通过省、国家评估验收，全面启动中职规范化建设。完善基本医疗卫生体系，加强公共卫生服务，推进市人民医院北城新区医院、肿瘤医院河东医疗区建设，办好省级区域医疗中心，抓好基层卫生综合改革试点，构建分级诊疗格局。完善全民健身体系，新建1000个农村健身工程，提升完善百里健身长廊，确保市民健身中心和市体校新校区建成启用，办好第四届中国沂河国际运动娱乐节。推动以人为本的城镇化。按照五年“三个100万人”的目标，扎实开展县域新型城镇化省级试点，有序推进农村人口市民化。完善管网设施，搞好重要路段排水设施和污水管网建设，推进西部热力中心和华能公司、金锣热电厂机组改造等热源项目和供热管网铺设，加快天然气“社区通”步伐；启动城市备用水源地规划建设，解决101万农村居民和1.3万农村学校师生的饮水安全问题。完善信息设施，实施城域网优化升级、光纤宽带接入网改造工程，建设智慧城市、数字城镇。继续开展城乡环境综合整治，建设一批生活垃圾处理设施，完成市垃圾填埋场提升改造工程，不断改善环境面貌，塑造城美乡美和“好山好水好风情、美丽乡村看沂蒙”的鲜明形象。提升交通出行条件。实施城乡综合交通规划。开通国际航班和全货机航线，增加客货吞吐量。构建中心城区骨干路网，建成江泉高架路，实施内环和中环道路、陶然路沂河桥、南京路沂河桥、西安路祊河桥等工程。继续推进农村公路改造，加快建设中心城区到各县和高速公路口的快速通道。完善公共交通设施，探索建设快速公交系统。加强社会治理创新。推进平安临沂建设，完善立体化社会治安防控体系；严格落实企业安全生产主体责任、政府部门监管责任和地方属地管理责任，强化隐患排查治理和安全预防控制，坚决遏制重特大安全事故；健全四级食品药品监管体系，提高监管能力，构建食品流通追溯体系；创新城市社区管理模式，深入推进村居干部经济责任审计，提升基层治理水平；发挥“五老”作用，做好关心下一代工作；继续稳定低生育水平，着力治理性别比偏高问题；健全自然灾害和突发事件应急机制，努力让广大群众拥有更安全的社会环境。

三、关于政府自身建设

各位代表，建设大临沂新临沂，实现“走在前列”的奋斗目标，前景美好，任重道远！必须大力弘扬沂蒙精神，大兴求真务实之风、开拓创新之风、清正廉洁之风，不断提高政府执行力和公信力，全面推进政府治理能力的大幅提升。

（一）坚持开拓奋进，打造有为政府。新常态，应有新作为。当前，我市正处于转型升级、改革攻坚、奋力赶超的关键时期。各级政府要敢于直面矛盾、勇于承担风险，提振精气神、聚集正能量，知难而进、迎难而上，抢占发展制高点、把握竞争主动权。要始终保持昂扬向上、奋发有为的精神状态，坚定必胜信念，抢抓机遇、用好机遇，咬定青山不放松，不达目的不罢休。要增强创新意识，善于用改革的办法破解难题，以新思维研究新问题、新理念落实新任务、新举措开创新局面。要树立“为官一任、造福一方”理念，坚持高标准，追求高境界，盯住先进找差距，敢与强手比高低，努力使各项工作创出经验、打造亮点、彰显特色。

（二）坚持立说立行，打造高效政府。按照重大任务明确分工、重点工作严格时限、决策部署督查落实、对懒政怠政严肃问责的要求，完善目标责任、执行监督和考核奖惩体系，切实提高各级政府执行力。对涉及经济社会发展全局的重点工作和重要事项，逐级分解细化，明确责任人，立下“军令状”，一件一件抓落实、一项一项求突破。充分发挥协同办公系统、电子监察平台的作用，加大效能监察力度，严格执行首问责任制、限时办结制，推动政府工作全面提速 金庸小说。着力解决言行不一、协作不力、推诿扯皮等问题，确保政令畅通，令行禁止。进一步改进文风会风，精简各类文件，压缩会议数量、规模和时间，少开会、开短会，少讲话、讲短话，腾出更多精力深入基层调查研究，摸实情、出实招、办实事。要按照客观规律办事，遵从群众意愿工作，积极为百姓多办实事，坚决防止盲目蛮干，坚决避免搞“花架子”，努力创造经得起历史和实践检验的业绩。

（三）坚持团结实干，打造诚信政府。严格落实“三严三实”要求，认真履行向社会作出的各项承诺，切实增强政府公信力，努力以政府诚信带动社会诚信。凡是党的群众路线教育实践活动中列出的整改事项，凡是确定的惠民实事、公布的标准时限、办理的诉求事项，凡是签订的合同书、协议书、责任书，都要一锤定音、一诺千金，决不“说空话、”“放空炮”。发挥政府诚信的导向作用，加强社会征信体系建设，搭建信用评估体系和公示平台，真正使守信者得益、失信者受罚。继续办好“12345”市民服务热线，严格落实领导干部直接联系和服务群众制度，真诚倾听群众呼声，真情关心群众疾苦，努力凝聚各方面齐心协力、干事创业的正能量。

（四）坚持从严治政，打造清廉政府。着力强化政府系统党组（党委）党风廉政建设主体责任，认真履行“一岗双责”，持续发力、形成常态。教育引导政府工作人员增强廉洁从政意识，敬畏权力、慎用权力，始终做到心中有党、心中有民、心中有责、心中有戒。全面落实中央八项规定和省、市委实施办法，从严控制行政经费和一般性支出，把更多财力用在保障改善民生上。坚持用制度管权、管事、管人，突出解决干部权力寻租和利益输送问题。加强财政资金分配使用、国有资产监管、政府投资采购、公共资源交易等重点领域、关键岗位的行政监察和审计监督，坚决防止管理监督失之于宽、失之于软的问题。着力正风肃纪，以零容忍态度纠正和查处损害群众利益的不正之风，全面推进政务规范、政事清明、政风廉洁，努力营造风清气正的良好环境。

各位代表！当前，临沂正处于重要的发展机遇期。作为革命老区，享受国家中部地区有关政策；作为区域中心城市，投资消费出口潜力巨大；作为人口面积大市，发展空间广阔；作为沂蒙精神发源地，全市上下风正气顺、心齐劲足，干事创业的热情高涨。只要我们坚定信心、奋发有为、团结实干，就一定能够保持经济行稳致远、社会文明和谐的良好局面。让我们在中共临沂市委的坚强领导下，站在新起点，抢抓新机遇，打造新优势，创造新业绩，为建设大临沂、新临沂，实现“走在前列”的宏伟目标而努力奋斗！
